# Dog importation and changes in canine intestinal nematode prevalence in Colorado, USA, 2013–2017

**DOI:** 10.1186/s13071-020-04283-z

**Published:** 2020-08-08

**Authors:** Jason Drake, Rudolph Parrish

**Affiliations:** Elanco Animal Health, 2500 Innovation Way, Greenfield, IN 46140 USA

**Keywords:** Relocation, Shelter, Animal rescue, Animal welfare, Roundworm, *Toxocara*, Hookworm, *Ancylostoma*, Whipworm, *Trichuris*, USA

## Abstract

**Background:**

Animal rescue and animal welfare organizations are relocating thousands of dogs per year following natural disasters and in attempts to provide greater adoption opportunities. Many dogs are sourced from the southeastern USA, which historically has a high prevalence rate for many parasites and parasitic diseases. The Colorado Department of Agriculture Pet Animal Care Facilities Act (PACFA) requires animal shelters and animal welfare organizations to report annually a variety of statistics including the numbers of dogs imported into Colorado from out of state. The Companion Animal Parasite Council (CAPC) provides data nationally, down to the state and county level, on a variety of common parasitic and vector borne diseases. These data make it possible to track changes in parasite prevalence over several years.

**Methods:**

Test results for canine roundworm, hookworm and whipworm were collected from the CAPC maps for 2013–2017. Dog importation data for 2014–2017 was collected from PACFA reports. For evaluation of the statistical significance of prevalence changes when comparing 2013 to 2017, 2 × 2 contingency tables were constructed with both positive and negative test results for each year and the data assessed using Chi-square tests to determine if the 2017 prevalence was significantly different than the 2013 prevalence for roundworm, hookworm and whipworm.

**Results:**

Significant increases in intestinal nematode prevalence occurred in Colorado from 2013 to 2017. The prevalence of canine roundworm rose 35.60%, the prevalence of canine hookworm rose 137.33% and the prevalence of canine whipworm rose 63.68%. From 2014 to 2017, over 114,000 dogs were transported into Colorado from out-of-state, by more than 130 animal shelters and rescue organizations. Three of the larger organizations reported that the majority of their dogs were obtained from New Mexico, Texas and Oklahoma. Texas and Oklahoma have historically much higher parasite prevalence than Colorado.

**Conclusions:**

Veterinarians in areas with historically low parasite prevalence where dogs from high parasite prevalence areas are arriving for adoption may need to reevaluate their recommendations regarding fecal examination and deworming frequencies as historic levels of intestinal parasite infection may no longer be accurate assessments of future infection risks.
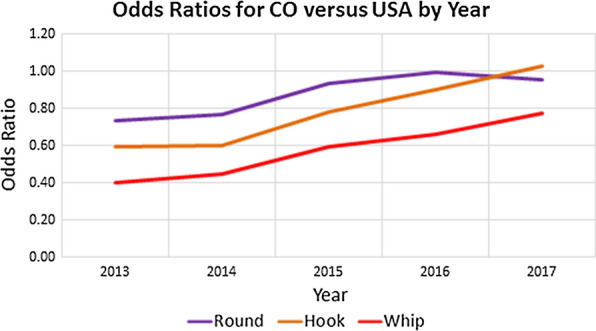

## Background

Animal rescue organizations in the USA move dogs interstate from states where high numbers of stray dogs are found into states where animal welfare organizations believe the dogs have a higher likelihood of being adopted. One organization, the ASPCA, actively seeks donations and awards relocation grants supporting animal transportation resulting in the shipment of more than 28,000 animals in 2017 alone [1]. A map on the ASPCA site shows New Mexico, Texas and Oklahoma among the “source areas” for animals and Colorado among the “destination areas” [1]. Animal rescue organizations actively provide rescue and relocation support during weather emergencies like floods and hurricanes, as well as movement of animals from areas with animal surplus to areas with perceived increased rehoming opportunities. A news article reported that nearly 30,000 dogs in one year were transported into Colorado, primarily sourcing dogs from the southern USA [2].

Infection with roundworms, hookworms and whipworms is common in dogs in animal shelters and in dogs rescued from hurricane and flooding disasters from the southern USA. National prevalence of these intestinal nematodes in shelters has ranged between 12.5–30.1%, with dogs in the southern USA having had the highest prevalence and dogs in the western USA having had the lowest prevalence [3, 4]. A recently published survey of animal rescue organizations reported that only 23.8% perform fecal testing/deworming prior to transporting dogs to a new facility [5].

The data utilized in the CAPC maps have been analyzed and reported in a number of different publications looking at a variety of situations from existing and previous parasite prevalence to forecasting of future parasite and vector-borne prevalence and risk [11–15].

A recent publication reported an increased prevalence of heartworms in dogs in Colorado from 2013–2017 of 67.5%, which correlated with importation of dogs from the southern USA and included dog importation data into Colorado by county 2013–2017 [11]. As a follow-up to this heartworm prevalence study, this additional study was conducted to evaluate the changes in intestinal nematode prevalence rates in relation to the documented importation of dogs into Colorado during the same period of time, 2013–2017.

## Methods

The Companion Animal Parasite Council (CAPC) has compiled data on canine intestinal nematodes based on test results compiled from two national diagnostic laboratories (ANTECH Diagnostics and IDEXX Laboratories). These laboratories conduct testing on samples provided to them by veterinary hospitals, then provide the test results to CAPC, representing approximately 30% of all fecal diagnostic tests performed in the USA, with results of more than six million canine fecal tests reported in 2017 alone [12, 13]. CAPC’s website includes both monthly and yearly results of fecal diagnostic testing for canine roundworm, hookworm and whipworm summarized nationally and at the state and county levels *via* interactive maps including data from 2012 to the present [13]. The data include total tests conducted, total tests positive for canine roundworm, hookworm and whipworm, and the percentage of tests positive at each geographic level (county, state and national). CAPC’s website states that data in the maps are a strong representation of parasite activity in the areas shown [12]. CAPC cautions that map data do not account for the total tests performed, as only a portion of all tests are reported to CAPC. Based upon such a large annual sample size, we consider the CAPC map data to be representative of the canine fecal testing and internal parasite prevalence rates in Colorado. Total canine fecal test results were obtained for Colorado from the counties for which results were reported in the CAPC maps. Using 2013 results as the baseline, changes in roundworm, hookworm and whipworm prevalence for each year from 2014 to 2017 were calculated. A linear regression model was employed to examine the relationship between Colorado prevalence and numbers of dogs imported. In addition, to examine the trend over the five-year period 2013–2017, odds ratios (ORs) for each parasite were computed that reflected the relative likelihoods of a positive test result for Colorado dogs compared to the US nationally.

Statistical data related to dog transportation into Colorado by over 130 animal rescue and shelter organizations were obtained from the Colorado Department of Agriculture Pet Animal Care Facilities Act (PACFA) on a yearly basis from 2014–2017 [14–17]. For evaluation of the statistical significance of prevalence changes when comparing 2013 to 2017, 2 × 2 contingency tables were constructed with both positive and negative test results for each year and the data assessed using Chi-square tests to determine if the 2017 prevalence was significantly different than the 2013 prevalence for roundworm, hookworm and whipworm. These were further confirmed via confidence intervals and significance tests on ORs.

Phone calls were made to some of the larger Colorado animal rescue and animal welfare organizations with the goal of gathering more details related to the states from where imported dogs originated.

Population statistics were obtained from the US Census Bureau to determine the total number of households within Colorado [18]. The total population of dogs in the state of Colorado was estimated utilizing an American Veterinary Medical Association (AVMA) formula provided within their 2012 U.S. Pet Ownership & Demographics Sourcebook as follows [19]: Number of dogs = 0.584 × Total number of households in the community.

## Results

Based upon data reported by the United States Census Bureau, there were 2,051,616 households in the state of Colorado 2012–2016 [18]. Based upon the number of households, and using the AVMA formulas for estimating dog populations, we calculated there were approximately 1,198,143 dogs in the state of Colorado in 2016.

According to the map data from CAPC, approximately 6.8 million fecal tests were performed on dog samples by ANTECH Diagnostics and IDEXX Laboratories in 2017. The estimated dog population in the USA in 2017 was approximately 70 million. Therefore, those fecal diagnostic tests indicated that 9.7% of dogs were tested for intestinal nematodes within the USA in 2017. In the state of Colorado, 127,732 fecal tests were reported for 2017 from an approximate population of 1.2 million dogs, representing 10.6% of dogs in Colorado tested for intestinal nematodes.

Dog importation PACFA reports showing sourcing of dogs from out-of-state was available from 37 of 64 counties within Colorado (58%) [11]. For the other 27 Colorado counties (42%), no dog importation data was available [11].

From 2014 through 2017, over 114,000 dogs were imported into 37 Colorado counties with 24,278, 28,147, 29,908 and 31,707 brought in from out-of-state in 2014, 2015, 2016 and 2017, respectively [11]. When considering the approximate Colorado dog population (1,198,143 dogs), the number of imported dogs documented represent roughly 2.0–2.6% of the entire Colorado dog population each year, assuming that all imported dogs survive and have been reported as required by all and animal welfare and shelter organizations throughout Colorado. Three larger organizations in the state of Colorado (Dumb Friends League, Humane Society of Pikes Peak, and Humane Society of Boulder Valley) did respond to requests for information regarding the original source of dogs imported into the state. Those data indicated that New Mexico was the primary source of imported dogs, accounting for approximately 30%, while almost half (49%) came from either Oklahoma or Texas. (Table 1).

**Table 1:** Top geographical sources of imported dogs

State	Count	Proportion (%)
NM	6488	30.32
TX	5638	26.35
OK	4883	22.82
CO	2194	10.25
KS	614	2.87
NE	459	2.15
LA	300	1.40

*Note:* 2017 imports reported by 3 of the larger organizations: Dumb Friends League, Humane Society of Pikes Peak and Humane Society of Boulder Valley

**Fig. 1:**
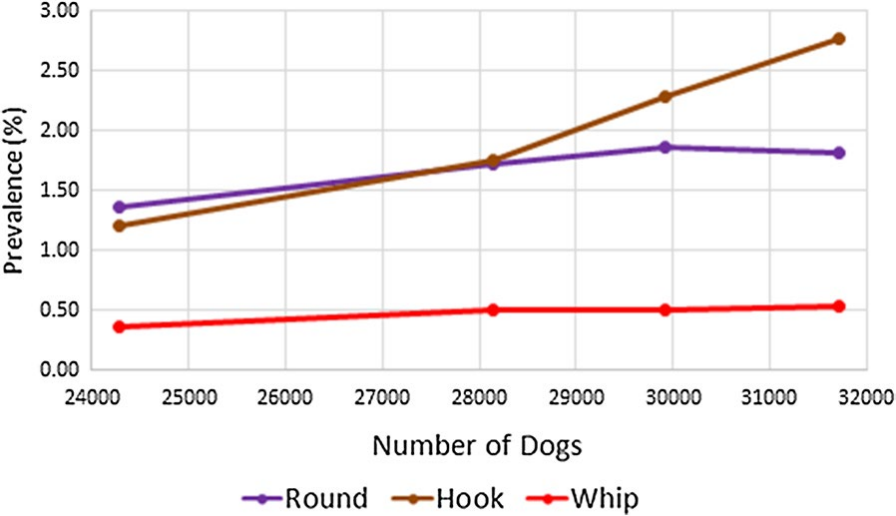
Prevalence (%) of roundworm, hookworm, and whipworm in Colorado plotted against the number of dogs imported during 2014–2017

**Fig. 2:**
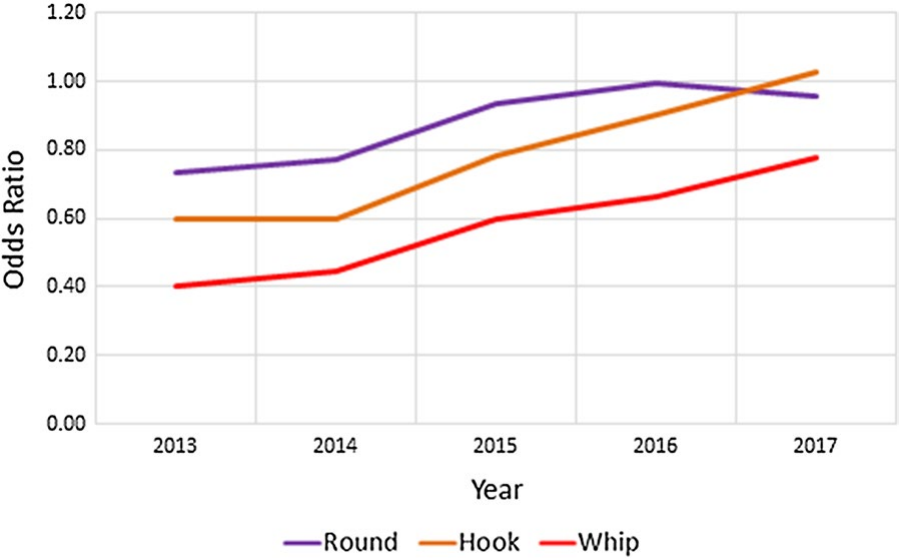
Odds ratios for roundworm, hookworm, and whipworm in Colorado relative to the USA for years 2013–2017

During 2014–2017, the total number of dogs reportedly imported into 37 counties within Colorado increased from 24,278 to 31,707 (30.6%) (Table 2). Based on the data from the CAPC maps, roundworm prevalence in Colorado in 2013 was 1.33%. This prevalence rose annually in 2014–2016 with prevalence estimates of 1.37%, 1.72% and 1.87%, respectively, dropping slightly to 1.81% in 2017 (Table 3). In 2017, the proportion of dogs positive for canine roundworm was significantly higher than in 2013 (Chi-square test: *χ*^2^ = 53.442, *df* = 1, *P* < 0.0001), with the 2017 prevalence of canine roundworm 35.60% higher than the 2013 prevalence. The data from the CAPC maps for hookworm prevalence in Colorado in 2013 was 1.17%. This prevalence rose annually in 2014–2017 with prevalence estimates of 1.20%, 1.75%, 2.29% and 2.77%, respectively (Table 4). In 2017, the proportion of dogs positive for canine hookworm was significantly higher than in 2013 (Chi-square test: *χ*^2^ = 448.249, *df* = 1, *P* < 0.0001), with the 2017 prevalence of canine hookworm 137.33% higher than the 2013 prevalence. Likewise, the data from the CAPC maps for whipworm prevalence in Colorado in 2013 was 0.33%. This prevalence also rose annually in 2014–2017 with prevalence estimates of 0.36%, 0.50%, 0.50% and 0.54%, respectively (Table 5). In 2017, the proportion of dogs positive for canine whipworm was significantly higher than in 2013 (Chi-square test: *χ*^2^ = 36.890, *df* = 1, *P* < 0.0001), with the 2017 prevalence of canine whipworm 63.68% higher than the 2013 prevalence. In linear regression of Colorado prevalence as a function of number of dogs imported over the years 2014–2017, increasing trends were observed for roundworm, hookworm, and whipworm (ANOVA for linear trend: *F*_(1, 2)_ = 12.63, *P* = 0.0708; *F*_(1, 2)_ = 55.74, *P* = 0.0175; *F*_(1, 2)_ = 22.50, *P* = 0.0417, respectively). As shown in Fig. 1, these were principally linear trends with associated *R*^2^ values of 0.863. 0.965, and 0.910, respectively. Odds ratios comparing the likelihood of a positive test for Colorado dogs compared to that for USA dogs across the five years, along with associated confidence intervals, are reported in Table 6 and illustrated in Fig. 2. These indicate that dogs from Colorado were much less likely than USA dogs to have tested positive for any of the nematodes in 2013, with values increasing annually to exhibit nearly the same odds in 2017: roundworm (0.73–0.96), hookworm (0.60–1.03), and whipworm (0.40–0.78).

**Table 2:** Imported dogs and percentage of estimated total in Colorado (1,198,143 estimated dogs in state)

Year	Dogs imported^a^	Percent increase from prior year	Percent of total dogs
2014	24,278	–	2.026
2015	28,147	15.94	2.349
2016	29,908	6.26	2.496
2017	31,707	6.02	2.646

^a^Based on totals from 37 counties

**Table 3:** Roundworm prevalence in Colorado and the USA by year

Year	Colorado		USA	
	Prevalence (%)	Percent change since 2013	Prevalence (%)	Percent change since 2013
2013	1.33	–	1.81	–
2014	1.37	2.67	1.77	− 2.12
2015	1.72	28.90	1.84	1.37
2016	1.87	40.38	1.88	3.75
2017	1.81	35.60	1.88	4.04

**Table 4:** Hookworm prevalence in Colorado and the USA by year

Year	Colorado		USA	
	Prevalence (%)	Percent change since 2013	Prevalence (%)	Percent change since 2013
2013	1.17	–	1.94	–
2014	1.20	2.41	1.98	1.72
2015	1.75	49.87	2.22	14.14
2016	2.29	96.51	2.54	30.43
2017	2.77	137.33	2.70	38.86

**Table 5:** Whipworm prevalence in Colorado and the USA by year

Year	Colorado		USA	
	Prevalence (%)	Percent change since 2013	Prevalence (%)	Percent change since 2013
2013	0.33	–	0.82	–
2014	0.36	9.06	0.80	− 2.24
2015	0.50	53.16	0.84	2.85
2016	0.50	53.53	0.76	− 6.77
2017	0.54	63.68	0.69	− 15.04

**Table 6:** Odds ratios (95% confidence intervals) for Colorado relative to the USA

Year	Roundworm	Hookworm	Whipworm
2013	0.73 (0.68–0.79)	0.60 (0.55–0.64)	0.40 (0.35–0.46)
2014	0.77 (0.72–0.82)	0.60 (0.56–0.64)	0.45 (0.39–0.51)
2015	0.93 (0.88–0.99)	0.78 (0.74–0.83)	0.60 (0.54–0.66)
2016	1.00 (0.95–1.04)	0.90 (0.86–0.94)	0.66 (0.60–0.73)
2017	0.96 (0.91–1.00)	1.03 (0.99–1.07)	0.78 (0.71–0.84)

## Discussion

Historically, Colorado has been an area of low prevalence for canine heartworm (*Dirofilaria immitis*) [11]. The prevalence of canine heartworms in Colorado was recently reported to have risen at greater than 3-times the USA national average from 2013–2017, rising from the 2013 prevalence of 0.5% to 0.84% in 2017, increasing 67.5% in only four years, during which time over 114,000 dogs were relocated to Colorado by animal shelters and animal rescue groups [11]. During this same time, the prevalence of canine roundworm rose 35.60%, the prevalence of canine hookworm rose 137.33% and the prevalence of canine whipworm rose 63.68%. These increasing rates of roundworm, hookworm and whipworm prevalence exhibited a trend similar to the increasing prevalence of canine heartworm during that same period, 2013–2017 [11]. This is further exemplified by the increasing odds ratios corresponding to the likelihoods of a positive test for dogs in Colorado relative to the USA for all three nematodes. As these odds ratios approach unity, there is effectively no significant difference in the odds for Colorado compared to the USA. For both roundworm and hookworm, the odds ratios are statistically the same by 2017 (i.e. 0.96 (95% CI: 0.91–1.00) and 1.03 (95% CI: 0.99–1.07), respectively). For whipworm, the trend is also in the same direction (OR: 0.78, 95% CI: 0.71–0.84) in 2017), and it is likely to converge to equivalence within a few years.

The data available from the CAPC maps only report total tests performed and total positive tests. The data do not report the age of the animals tested. Shifting demographics to younger animals tested could explain increases in roundworms, which are seen more often in young animals. However, a shift to a younger population of animals should also result in a drop in whipworm prevalence, as whipworms are not as common in young animals [11]. Because whipworms are also increasing in prevalence, a shift in the demographics to a younger population is unlikely to be an explanation for the increases in gastrointestinal nematode prevalence.

Only about 24% of animal shelters and rescue organizations perform fecal testing/deworming prior to transporting dogs to a new facility [5]. Over 114,000 dogs were transported into Colorado from out-of-state, by more than 130 animal shelters and rescue groups, from 2014 to 2017. Based on the scale of dog movement demonstrated, veterinarians and pet owners in geographies with historically low parasite prevalence receiving similar shipments of dogs from areas with higher parasite prevalence may not be adequately testing and treating dogs for emerging parasitic risks.

The data obtained from PACFA and CAPC show clear correlation between dog importation and rising prevalence in gastrointestinal nematodes. These changes are similar to the change in heartworm prevalence previously published [11]. However, limitations of the data currently do not allow for examination of other factors which could also be responsible for increases seen in the prevalence of these gastrointestinal nematodes. Examples of limiting factors which could also increase parasite prevalence include a decrease in the age of dogs tested or increased social activities such as dog day care and dog park visitation.

Data obtained from three of the larger organizations, which were importing dogs into Colorado, showed that nearly half of the dogs had originated from either Texas or Oklahoma (Table 2). Based upon the data shown on the CAPC maps for 2017, 2.28%, 6.57% and 1.27% of dogs in Oklahoma and 1.34%, 4.48% and 0.57% of dogs in Texas tested positive for roundworm, hookworm and whipworm, respectively [13]. The prevalence of roundworm and whipworm in Oklahoma in 2017 was 2-fold higher than Colorado, while the prevalence of hookworms in Oklahoma and Texas was 2–3-fold higher than the prevalence of hookworm in Colorado in 2017 [13].

## Conclusions

Veterinarians in areas with historically low parasite prevalence where dogs from high parasite prevalence areas are arriving for adoption may need to reevaluate their recommendations regarding fecal examination and deworming frequencies as historic levels of intestinal parasite infection may no longer be accurate assessments of future infection risks. Prevalence of canine roundworms, hookworms and whipworms in Colorado increased 35.60%, 137.33% and 63.68%, respectively, from 2013–2017. Because of this dog relocation trend across the USA, pet owners and veterinarians in historically low parasite prevalence areas should consider reevaluating the risks posed by a variety of parasitic and vector borne diseases and should reinforce the adherence to prevention and testing guidelines provided by the Companion Animal Parasite Council and the American Heartworm Society [20, 21]. While animal welfare organizations and animal shelters provide important services like rescue and re-homing, it is likely that relocated dogs are increasing the prevalence and local infection risks of a wide variety of parasitic diseases. Further research related to the intestinal nematode infection status of imported dogs would help confirm this association of increased intestinal nematode prevalence and the relocation of dogs.

## Data Availability

Data supporting the conclusions of this article are provided within the article. The heartworm testing datasets analyzed during the present study are available in the CAPC Prevalence Maps, https://www.capcvet.org/maps. The Colorado dog importation datasets analyzed during the present study are only available for 3 years from the Colorado Department of Agriculture Pet Animal Care Facilities Act Program, https://data.colorado.gov/.

## Abbreviations

AVMA: American Veterinary Medical Association
CAPC: Companion Animal Parasite Council
PACFA: Pet Animal Care Facilities Act
SE: Standard error
CI: Confidence interval
